# Evaluation of structurally different benzimidazoles as priming agents, plant defence activators and growth enhancers in wheat

**DOI:** 10.1186/s13065-019-0546-2

**Published:** 2019-03-11

**Authors:** Arruje Hameed, Amjad Hameed, Tahir Farooq, Razia Noreen, Sadia Javed, Shaheera Batool, Ashfaq Ahmad, Tahsin Gulzar, Matloob Ahmad

**Affiliations:** 1 Department of Biochemistry, Government College University, Faisalabad, Pakistan; 2 Nuclear Institute for Agriculture and Biology (NIAB), Jhang Road, P.O. Box 128, Faisalabad, Pakistan; 3 Department of Applied Chemistry, Government College University, Faisalabad, Pakistan; 4 Department of Biochemistry, Multan Institute of Health Sciences, Multan, Pakistan; 5 Department of Chemistry, Government College University, Faisalabad, Pakistan

**Keywords:** Seed priming, Antioxidants, Benzimidazole, Hydrolytic enzymes, Wheat

## Abstract

Priming is a valuable, facile and well-established technique used to enhance seed quality to achieve rapid germination, establishment of stress resistance and improvement of crop yields. Different natural and synthetic priming agents have been used for better crop performance and abiotic stress management. In this study, four different benzimidazoles were selected as priming agents and their comparative effects were evaluated on different biochemical attributes including total soluble protein, total oxidant status, MDA contents, antioxidant enzymes (SOD, POD) and hydrolytic enzymes (protease, estrases) compared to control. Treatments with 2-thio-1-*H*-benzimidazole reduced total soluble proteins and increased total oxidant status significantly but no considerable effect was observed on other parameters. Priming with 2-(4-chlorophenyl)-1-*H*-benzimidazole considerably increased the total oxidant status and a little improvement was observed in total soluble proteins. Seeds primed with 1-*H*-benzimidazole showed a noticeable decrease in the protease activity while all other priming treatments were unable to induce any detectable change compared to control. The treatment with 2-(4-methoxyphenyl)-1-*H*-benzimidazole induced maximum reduction in MDA contents and POD activity. Moreover, all benzimidazole priming treatments reduced mean germination time, increased germination percentage and germination rate of wheat seeds.

## Introduction

Due to rising global population, it has been estimated that the demand for wheat is going to be doubled in 2050 [1]. To satisfy these rising wheat demands, farmers are supposed to boost crop yields by adopting new farming strategies. In this context, enhanced seed qualities has become priority requirements to achieve uniform and rapid seedling emergence for better crop performance and finally increased yield [2]. Seed quality is enhanced by employing facile, easily practicable and well established treatment called priming [3]. As a result of priming treatments, germination rate increases with the development of high level stress tolerance which enhances crop yields [4]. In fact, priming induces pre-germinative metabolism to various level in seeds depending upon their species, physiology and morphology [5]. These specific metabolic changes trigger ATP production, de-novo synthesis of proteins and nucleic acids, activation of antioxidant enzymes and DNA repair, accumulations of phospholipids and sterols [6, 7]. The activation of these cellular mechanisms protect genome integrity, ensure rapid germination with fast seedling emergence thus help to provide high crop yields [8].

Around the globe wheat is the major cereal crop fulfilling almost half of the protein requirements and feeds at least one-third world population. Often wheat crop productivity is limited by slow germination rate, reduced seedling vigor, slow growth and development rates under normal and stress conditions [9]. Under such situations, various natural and synthetic chemicals have been used as priming agents for various crops including wheat. Chemical priming offers effective opportunities for crop stress managements as it induces significant tolerance against a range of abiotic stresses [10]. On-farm priming of wheat seeds with ascorbic acid, salicylic acid, auxins, H_2_O_2_, polyethylene glycol, kinetin and GA_3_ etc. has been reported to improve aforementioned germination, seedling growth, non-enzymatic and enzymatic antioxidants related attributes leading to high grain yield [3].

The benzimidazole and its derivatives are exceptional structural motif of wide interest exhibiting a broad spectrum of applications across a range of scientific disciplines [11–13]. The benzimidazole nucleus with varied substituents has proved as a privileged moiety with diverse potential of clinical and biological activities including antiviral, antibacterial, anti-tumor, anti-hypertensive, anti-diabetic and anti-HIV etc. [14, 15]. Compounds incorporating benzimidazole have also been used as agrochemicals with fungicidic and plant growth regulating properties [16]. Further, they provide protection and insulate plants against various environmental stresses [17]. Mangnucka et al. treated rye grains with 10 ppm of carbendazim and benomyl before they were allowed to germinate for 5 days [18]. These benzimiazole-based fungicides greatly affected the biosynthesis of resorcinol and fresh and dry biomass of seedlings under thermal and light growth conditions. Seed treatments with Ambiol^®^, a known benzimidazole-based antioxidant increased germination, enhanced growth and improved stress tolerance in seedlings of many species [19–21]. Tomato seed treatments with Ambiol induced positive effects on germination, growth and seedling development which were passed-on to next generation. Vital parameters like photosynthesis, leaf area, percent germination, root mass and shoot mass were considerably improved in parents as well as in progeny [22].

In this study four different benzimidazoles were selected as wheat seed priming agents and their effects on biochemical attributes were evaluated. The subsequent sections do explain the comparative effects of these benzimidazoles on vital biochemical and germination parameters.

## Materials and methods

### Chemistry

**Fig. 1:**
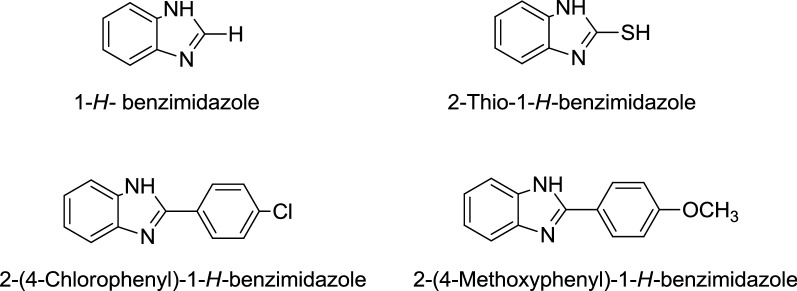
Structurally different benzimidazoles selected as priming agents

Following known benzimidazoles were selected as priming agents for wheat seeds (Fig. 1) [23].

### Seed collection and priming

For this priming study, the spring wheat (*Triticumaestivum* L. cv. GLAXY-2013) seeds were obtained from Wheat Section, Nuclear Institute of Agriculture and Biology (NIAB), Faisalabad, Pakistan. Wheat seed priming was achieved by soaking them in aerated solutions of four different benzimidazoles with 20 and 30 ppm concentrations for 8 h. Afterwards, they were washed and dried under shade at 26 ± 2 °C until they gained original weight. Separately, seeds were soaked in distilled water for 8 h to achieve hydro-priming. Untreated or non-primed seeds were used as control for comparison in biochemical analyses and germination studies.

### Biochemical analysis and germination studies

Different biochemical parameters were analyzed in primed, hydro-primed and non-primed wheat seeds to evaluate the effects of benzimidazole priming treatments. According to well-established methods for estimation and extraction of enzymes and other biochemical parameters, hydro-primed, primed and non-primed seeds were grounded using 50 mM potassium phosphate buffer with pH 7.4. At 4 °C, the grounded material was put on centrifugation at 15,000×*g* for 20 min and the supernatant was used for quantification studies of different enzymes. The method described by Bradford was followed for protein estimation in seed samples [24]. Total oxidant status was determined by following the method presented by Erel et al. [25]. This method estimates the presence of oxidants which oxidize Fe^+2^ to Fe^+3^. The method presented by Giannopolitis and Ries was followed with little modification to determine superoxide dismutase (SOD) activities [26]. The method initially presented by Heath and Packer and then modified by Dhindsa et al. and Zhang and Kirkham was used to determine malondialdehyde (MDA) contents [27–29]. The method of Drapeau was followed for protease activity determination [30]. The method developed by Chance and Maehly was employed for the determination of peroxidase (POD) activities [31]. The enzyme activities were expressed on seed weight basis. According to the methods of Van Asperen [32], the α-naphthyl acetate and β-naphthyl acetate were used as substrates for the determination of α-esterases and β-esterases [33].

**Fig. 2:**
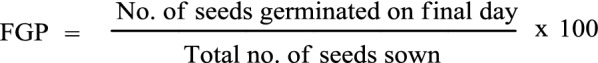
Calculation of % germination

Germination potential of the primed and control wheat seeds was estimated. To test seed germination and seedling vigor under osmotic stress, four replicates of 25 seeds were germinated in 12 cm diameter petri dishes at 25 °C. A seed was scored as germinated when coleoptile and radicle lengths reached 2–3 mm. Counts of germinating seeds were made twice a day at different time intervals (20, 28, 44, 52, 68, 76, 92 and 100 h), starting on the first day of imbibition, and terminated when maximum germination was achieved. Final germination percentage was measured according to following formula (Fig. 2).

Mean germination time (MGT) was calculated as following [34],

$${\text{MGT}}\, = \,{{\sum {\text{Dn}} } \mathord{\left/ {\vphantom {{\sum {\text{Dn}} } {\sum {\text{n}} }}} \right. \kern-0pt} {\sum {\text{n}} }}$$

Germination index (GI) was calculated as described in the Association of official Seed Analysts (AOSA) and the energy of germination was recorded according to a well-known method [35, 36].

### Statistical analysis

The recorded data was analyzed statistically by applying descriptive statistics. The significance between means was measured using Tucky’s test at 5% probability level using XL-STAT. Values presented are mean ± SD with different alphabets differ significantly from each other.

## Results and discussions

**Fig. 3:**
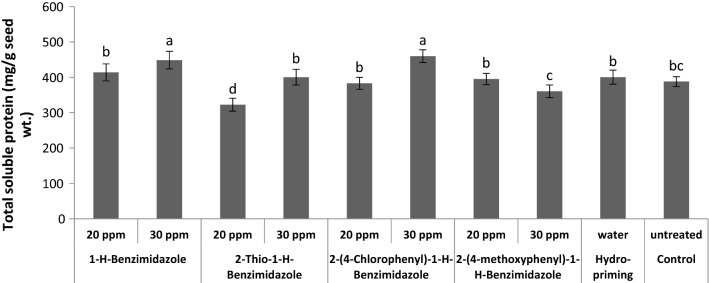
Effect of different seed priming treatments on total soluble protein contents in wheat seeds

Changes in the total soluble protein contents in non-primed, hydro-primed and benzimidazole primed wheat seeds were measured (Fig. 3). A noticeable improvement in the protein contents was observed in the seeds primed with 30 ppm of both 1-*H*-benzimidazole and 2-(4-chlorophenyl)-1-*H*-benzimidazole. While priming with 20 ppm of 2-thio-1-*H*-benzimidazole reduced total soluble proteins to some extent compared to control. However, all other treatments showed no apparent difference in protein contents compared to control. It may be suggested that the priming with benzimidazoles did not interrupt the cellular pathways or related enzymes involved in the biosynthesis of proteins. Jafar et al. reported an increase in total soluble proteins when wheat seeds were primed with salicylicate, kinetin, CaCl_2_ and ascorbate [37]. Similarly, Bajwa et al. also reported an increase in total soluble proteins when benzyl amino purine was used as a priming agent for wheat seeds [38].

**Fig. 4:**
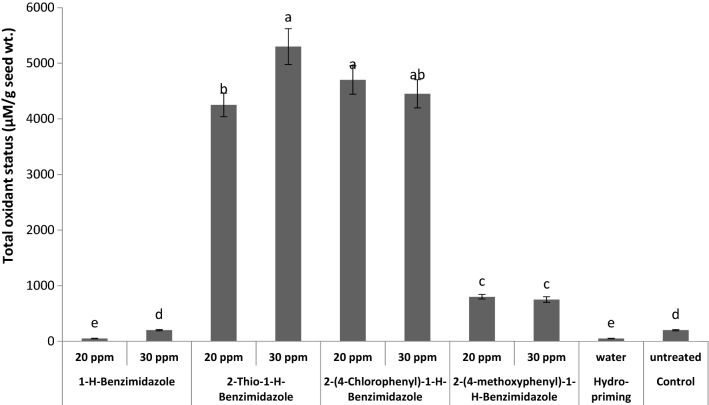
Effect of different seed priming treatments on total oxidant status in wheat seeds

Effects of different benzimidazole seed priming treatments on total oxidant status in wheat seeds were evaluated (Fig. 4). Total oxidant status increased remarkably in seeds primed with 20 ppm 2-thio-1-*H*-benzimidazole and 30 ppm 2-(4-chlorophenyl)-1-*H*-benzimidazole as compared to untreated control seeds. While a noticeable decrease in total oxidant status was observed as a result of 20 ppm 1-*H*-benzimidazole and hydro-priming. The oxidants were long considered as damaging species for germinating seeds. Recent studies have confirmed their well-established functions in cell signalling, regulation of gene expressions and mobilization of reserves during seed germination [39]. In germinating seeds the metabolically active compartments like mitochondria (for respiratory activities), plasma membrane (by NADPH oxidase) glyoxysomes (for lipid catabolism), peroxisomes (for purine catabolism) become main source of oxidants production. Strong increase in respiratory activities with enhanced production of oxidants are associated with germination [40, 41]. The aforementioned benzimiazole treatments which increased total oxidants significantly might have accelerated the metabolic activities to boost seed germination. It has also been confirmed from the fast germination rate during the first 24 h as shown in (Fig. 14).

**Fig. 5:**
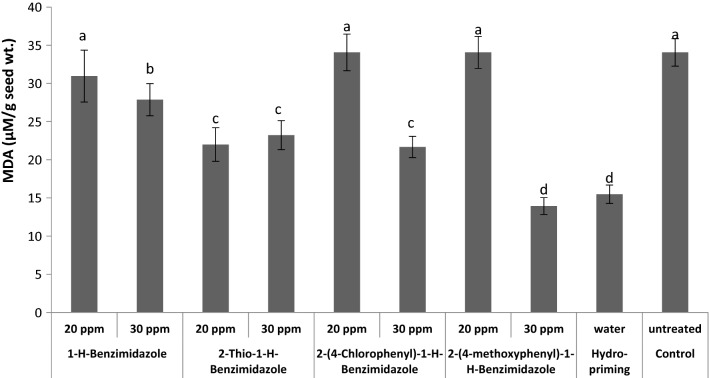
Effect of different seed priming treatments on MDA content in wheat seeds

During this wheat seed priming study, the level of lipid peroxidation in seeds was measured in terms of MDA contents (Fig. 5) [42, 43]. Priming with 20 ppm of 1-*H*-benzaimidazole, 2-(4-chlorophenyl)-1-*H*-benzimidazole and 2-(4-methoxyphenyl)-1-*H*-benzimidazole showed no observable difference in MDA contents as compared to control. Whereas, all other treatments showed a significant reduction in the MDA contents as compared to control. The treatment with 30 ppm 2-(4-methoxyphenyl)-1-*H*-benzimidazole induced maximum reduction in MDA contents. The MDA contents are considered as indicator of lipid peroxidation caused by reactive oxygen species (ROS).

The ROS are toxic by-products of aerobic metabolism and results in oxidative stress. The oxidative stress cases destruction of biomolecules like lipid, proteins, DNA and also inactivates antioxidant enzymes [44]. Reduction in MDA level represents low levels of oxidative stress while high levels of MDA suggest overproduction of fatal free radicals [45, 46]. It may be concluded that seed priming with 30 ppm 2-(4-methoxyphenyl)-1-*H*-benzimidazole reduced ROS levels and oxidative stress in wheat. Wheat seed priming with polyethylene glycol has been reported to reduce MDA contents [47]. Recently, priming treatments with mercapto-triazoles also reduced MDA content in wheat seeds representing a reduction in oxidative stress [48].

**Fig. 6:**
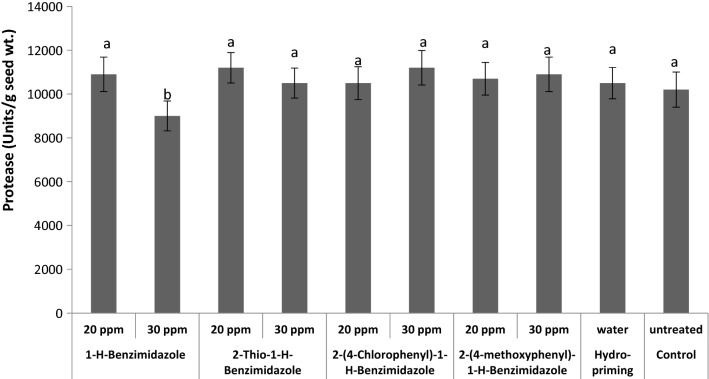
Effect of different seed priming treatments on protease activity in wheat seeds

The changes in protease activity in hydro-primed, benzimidazole primed and control wheat seed were also examined (Fig. 6). Seeds primed with 30 ppm of 1-*H*-benzimidazole showed a perceptible decrease in the protease activity while all other priming treatments were unable to induce any detectable change compared to control. No change in protease activity suggests that the proteins are in un-hydrolysed form in seeds primed with benzimidazoles. It is also confirmed by the unchanged contents of the total soluble proteins shown in Fig. 2 [49].

**Fig. 7:**
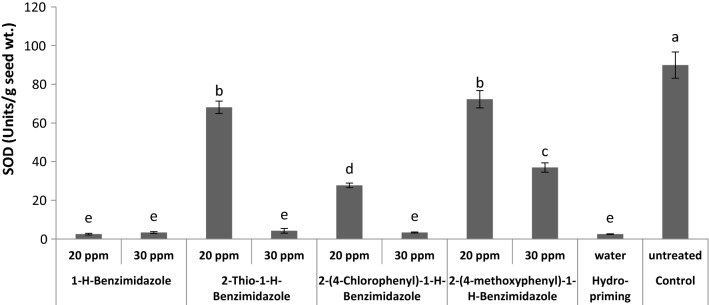
Effect of different seed priming treatments on SOD activity in wheat seeds

Treatment with 20 ppm 2-thio-1-*H*-benzimidazole and 2-(4-methoxyphenyl)-1-*H*-benzimidazole induced an observable decrease in SOD compared to control. Priming with both levels of 1-*H*-benzimidazole and 30 ppm of both 2-thio-1-*H*-benzimidazole and 2-(4-chlorophenyl)-1-*H*-benzimidazole presented maximum decrease in SOD activity compared to control (Fig. 7). Previously, it has been reported that the different combinations of chemical and hormonal treatments increased SOD activity in wheat seeds [50]. Wheat seed priming with chitosan and sodium nitroprusside (SNP) have also been reported to increase SOD activity [51, 52]. The SOD acts as a first line of defence against oxidative stress as these metalloenzymes catalyse dismutation of superoxide radicals to oxygen and hydrogen peroxide [53].

**Fig. 8:**
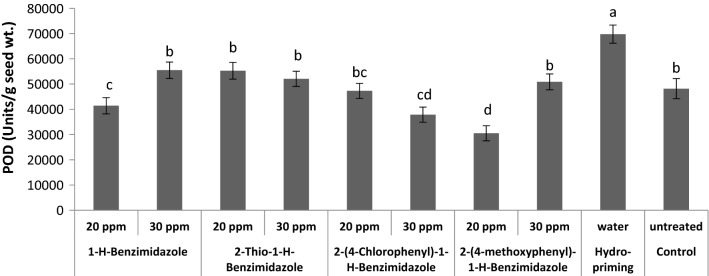
Effect of different seed priming treatments on peroxidase activity in wheat seeds

A significant decrease in POD activity was observed in seeds primed with 20 ppm 1-*H*-benzimidazole, 20 and 30 ppm 2-(4-chlorophenyl)-1-*H*-benzimidazole compared to control. Also, priming with 20 ppm 2-(4-methoxyphenyl)-1-*H*-benzimidazole decreased the POD (Fig. 8). However, no perceptible change in POD was recorded as a result of treatments with 2-thio-1-*H*-benzimidazole. The POD helps in scavenging reactive oxygen species which otherwise could cause oxidative injury [54]. The down regulation of POD suggests its fewer requirements with parallel low production of ROS in primed seeds. From the decreased SOD and POD levels in primed seeds, it could be presumed that benzimidazole treatments have protected the wheat seeds from oxidative stress. In our previous studies, a decrease in POD activity was also recorded when wheat seeds were primed with 10, 15 and 20 ppm of four structurally different triazoles [48].

**Fig. 9:**
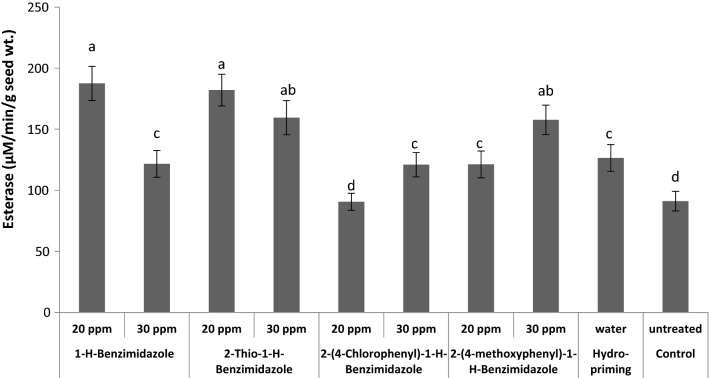
Effect of different seed priming treatments on esterase activity in wheat seeds

Except 20 ppm 2-(4-chlorophenyl)-1-*H*-benzimidazole all other priming treatments significantly increased the esterase activity compared to control (Fig. 9). The maximum boost in esterase activity was induced as a result of priming with 20 ppm of both 1-*H*-benzimidazole and 2-thio-1-*H*-benzimidazole. The treatment with 20 ppm 2-(4-methoxyphenyl)-1-*H*-benzimidazole and 30 ppm of both 1-*H*- benzimidazole and 2-(4-chlorophenyl)-1-*H*-benzimidazole increased esterase activity equivalent to hydro-priming. The increased activity of estrases represents accelerated metabolic processes in germinating wheat seeds. Indirectly, it has also been confirmed from high level of total oxidants and low contents of MDA. Increase in esterase activity was also observed when wheat seeds were primed with SNP as reported by Hameed et al. [52].

**Fig. 10:**
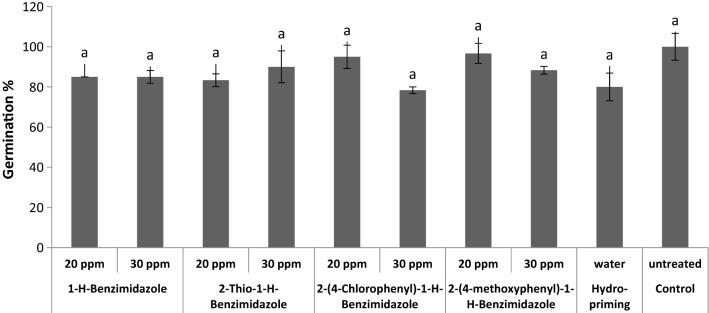
Effect of benzimidazole priming on final germination %

Further, the benzimidazole priming effects on wheat seed germination parameters were also evaluated. All priming treatments showed no significant effect on germination percentage of wheat seeds as compared to control (Fig. 10). However, preconditioning of tomato seeds with Ambiol were reported to increase germination percentage by 12.4% [22]. Other literature reports suggests that wheat seed priming with triazolic compounds, hormones and sodium nitroprusside induced an increase in percentage germination [48, 52, 55].

**Fig. 11:**
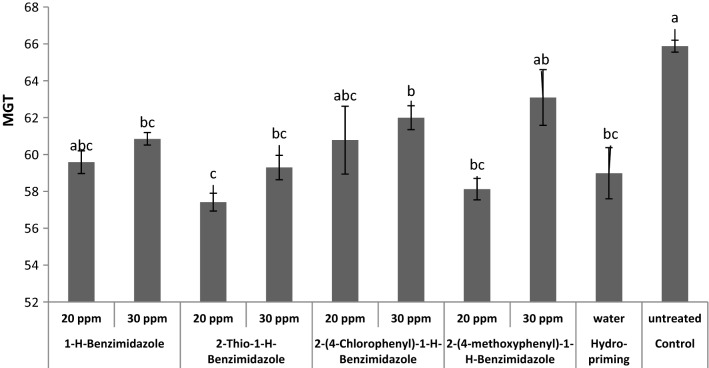
Effect of benzimidazole priming on mean germination time (h) of wheat seeds

All benzimidazole treatments decreased the mean germination time (MGT) of wheat seeds as compared to control seeds (Fig. 11). Hydro-priming also effectively decreased the MGT of seeds. The shortest mean germination time with most rapid germination was observed in seeds treated with 20 ppm of 2-thio-1-*H*-benzimidazole and proved the best priming treatment in this regard. It has been reported that wheat seed priming with SNP also reduced GMT [52]. Preconditioning of tomato seeds with Ambiol also significantly reduced MGT [22].

**Fig. 12:**
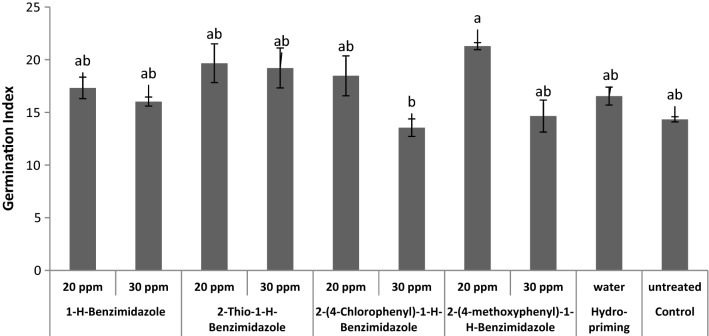
Effect of benzimidazole priming on germination index of wheat seeds

The effects of benzimidazole priming on wheat seed germination index were also evaluated (Fig. 12). The results showed that benzimidazole treatments increased the germination index of wheat seeds. A significant increase in germination index was induced by 20 ppm 2-(4-methoxyphenyl)-1-*H*-benzimidazole priming treatment. Wheat seed priming with differently substituted triazoles also reported to improve germination rate and germination index [48].

**Fig. 13:**
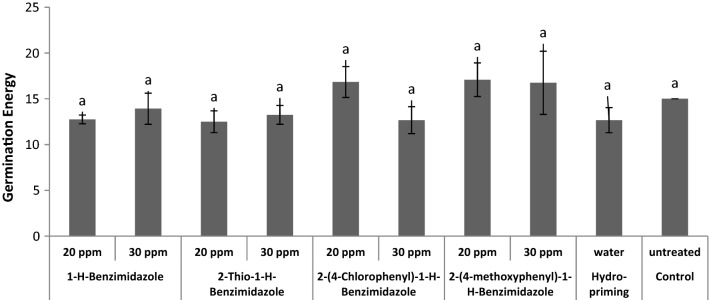
Effect of benzimidazole priming on germination energy of wheat seeds

Effects of benzimidazole priming were also evaluated on wheat seed germination energy (Fig. 13). All priming treatments showed no significance effect on germination energy as compared to control.

**Fig. 14:**
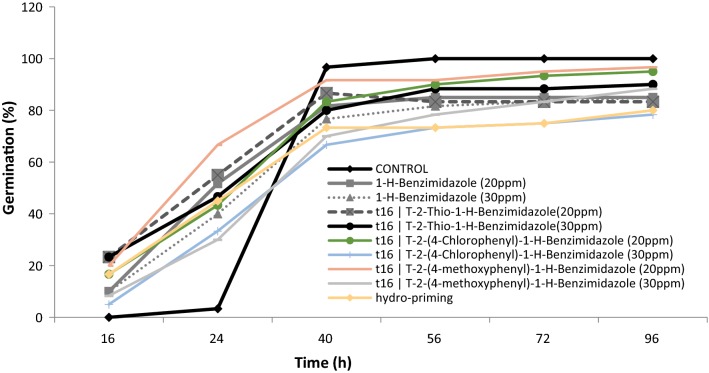
Effect of benzimidazole priming, hydro-priming and non-priming on germination rate of wheat seeds

Effect of benzimidazole treatments on germination rate was observed. All benzimidazole treatments induced early germination during first 24 h when the control seeds were not germinating at all (Fig. 14). Previously, it has also been observed that priming with triazolic compounds, hormones and SNP increased germination rate in wheat seed [48, 52].

## Conclusions

In conclusion, differently substituted benzimidazoles induced different effects on each biochemical parameters. Treatments with 20 ppm 2-thio-1-*H*-benzimidazole reduced total soluble proteins and increased total oxidant status significantly. Priming with 30 ppm 2-(4-chlorophenyl)-1-*H*-benzimidazole considerably increased total oxidant status and a little improvement was observed in total soluble proteins whereas treatment with its 20 ppm did not affect esterase activity. Seeds primed with 30 ppm of 1-*H*-benzimidazole showed a perceptible decrease in the protease activity while all other priming treatments were unable to induce any detectable change compared to control. The treatment with 30 ppm 2-(4-methoxyphenyl)-1-*H*-benzimidazole induced maximum reduction in MDA contents and priming with its 20 ppm decreased POD activity. All benzimidazole priming treatments reduced mean germination time, increased germination percentage and germination rate of wheat seeds and have numerous potential to be used as germination enhances under normal and stressed conditions.

## Abbreviations

SOD: superoxide dismutase
MDA: malondialdehyde
POD: peroxidase
ROS: reactive oxygen species
MGT: mean germination time
GI: germination index
SNP: sodium nitroprusside
